# The chain-mediating roles of social support and depression in the relationship between tuberculosis knowledge and self-management: a cross-sectional study based on the ABC-X model

**DOI:** 10.3389/fpubh.2025.1640626

**Published:** 2025-10-22

**Authors:** Chune Gu, Bofei Liu, Huijuan Wang, Yaling Ma, Lei Guo

**Affiliations:** ^1^Department of Nursing, The Fourth People's Hospital of Ningxia Hui Autonomous Region, Yinchuan, China; ^2^Department of Tuberculosis, The Fourth People's Hospital of Ningxia Hui Autonomous Region, Yinchuan, China

**Keywords:** tuberculosis, self-management, disease knowledge, social support, depression

## Abstract

**Background and objective:**

Self-management plays a vital role in tuberculosis (TB) care, yet remains suboptimal among patients due to limited disease knowledge and psychosocial challenges. While prior studies have explored the individual roles of knowledge, social support, or depression in TB treatment, few have examined their interactive and sequential effects. This study aimed to investigate whether perceived social support and depression mediate the relationship between TB knowledge and self-management, using the ABC-X family stress model as the theoretical framework.

**Methods:**

A cross-sectional survey was conducted. Using convenience sampling to select 204 patients with primary pulmonary TB hospitalized at a tertiary TB hospital in Ningxia, China. Participants completed validated questionnaires assessing TB knowledge, perceived social support, depression, and self-management ability. Correlation analysis was used to assess associations among key variables. Mediation analysis was performed using SPSS PROCESS macro (Model 6), with 5,000 bootstrap resamples to estimate direct and indirect effects.

**Results:**

TB knowledge was positively correlated with self-management and perceived social support, and negatively correlated with depression. Perceived social support was negatively correlated with depression and positively correlated with self-management. Depression was negatively correlated with self-management. The chain-mediating path—TB knowledge → social support → depression → self-management—was statistically significant and accounted for 5.00% of the total effect, while the total indirect effect explained 42.74% of the total effect.

**Conclusion:**

This study demonstrated that TB knowledge significantly influences self-management, both directly and indirectly, through the sequential mediating roles of perceived social support and depression. Enhancing TB knowledge alone may be insufficient without concurrently addressing social support and mental health. Multidimensional interventions integrating education, social support enhancement, and psychological care are essential for improving patient adherence and outcomes. The ABC-X model provides a useful framework for guiding future TB self-management interventions.

## 1 Introduction

Tuberculosis (TB) remains a major cause of morbidity and mortality worldwide. In 2022, the World Health Organization (WHO) reported 10.6 million new TB cases and 1.3 million TB-related deaths, making it the second most deadly infectious disease after COVID-19 (1). China bears a significant portion of the global burden, accounting for approximately 748,000 new cases annually and ranking third among the 30 high-burden TB countries (2). Despite the implementation of the DOTS strategy and steady global progress, TB continues to impose a substantial health, economic, and social burden, particularly in resource-limited settings (3).

To enhance TB control, the WHO has advocated for more person-centered strategies, with self-management emerging as a critical complementary approach to DOTS (4). Self-management encompasses patients' active involvement in disease monitoring, symptom control, treatment adherence, and psychosocial adjustment (5, 6), and is associated with improved clinical outcomes and reduced recurrence (7–9). However, real-world studies show that TB self-management remains insufficient, especially among newly diagnosed patients, due to low health literacy, limited social support, and untreated psychological distress (10, 11). Patients with poor self-management exhibit lower treatment adherence, with an incidence rate of 33%−50% significantly increasing the risk of drug resistance, relapse, and death (8, 12–14).

Although previous research has explored individual influences on TB self-management—such as knowledge, social support, or depression—most studies have addressed these factors in isolation (4, 15–18). The potential interactions and sequential mechanisms by which these psychosocial variables jointly influence behavior are underreported. In particular, it remains unclear how TB knowledge shapes self-management behavior through perceived social support and psychological wellbeing. Depression is prevalent among TB patients, exacerbated by disease stigma, prolonged treatment, and adverse drug effects (19–23). While in other chronic disease contexts, chain mediation pathways involving knowledge, support, and emotion have been proposed (24–27), this has not been systematically studied in TB populations. Furthermore, few studies have employed established theoretical frameworks to explain these mechanisms, especially within the context of TB.

To address these gaps, this study applies the ABC-X family stress model (28) to investigate the relationship between TB knowledge and self-management, with perceived social support and depression as chain mediators. We hypothesize that TB knowledge enhances self-management both directly and indirectly by increasing perceived social support and reducing depression. By clarifying these pathways, our aim is to provide theoretical and empirical support for developing comprehensive, multidimensional intervention strategies that improve self-management in TB patients and support successful treatment completion.

## 2 Methods

### 2.1 Study design and participants

This was a cross-sectional study conducted in accordance with the STROBE guidelines. Using convenience sampling, patients hospitalized in the Department of Respiratory Medicine at a tertiary TB hospital in Ningxia between December 2023 and February 2024. This hospital is the only tertiary-level TB specialty center in the region, admitting and managing patients with TB from across Ningxia.

Eligibility criteria included: (1) confirmed diagnosis of primary pulmonary TB according to WS288–2017 guidelines (29); (2) aged 18–75 years; (3) expected to receive standard anti-TB therapy for at least 6 months; and (4) ability and willingness to provide informed consent. Exclusion criteria were: (1) co-infection with HIV, hepatitis B, or other major infectious diseases; (2) severe comorbidities including heart, liver, kidney, or brain failure; (3) hemoptysis or unstable clinical condition; and (4) individuals who are unable to communicate due to severe hearing or visual impairments, mental disorders, etc.

### 2.2 Sample size calculation

The required sample size was estimated using the formula for continuous variable analysis: N = (Uα × S/δ)^2^, where α = 0.05 (Uα = 1.96), S = 25.00 (standard deviation from pilot data on self-management), and δ = 3.7 (permissible error) (30). The minimum calculated sample size was 175. After accounting for a 15% rate of invalid responses, the final target sample was adjusted to 201. A total of 211 questionnaires were distributed, and 204 valid responses were collected, yielding a valid response rate of 96.7%.

### 2.3 Ethical considerations

Ethical approval for this study was granted by the Ethics Committee of the Fourth People's Hospital of Ningxia Hui Autonomous Region (Approval No. 2024-N180). All participants provided written informed consent prior to enrollment. Confidentiality and anonymity were maintained throughout the study.

### 2.4 Data collection

Data was collected by the first author using paper questionnaires distributed on-site within the first week after the patient's diagnosis and hospitalization. Eligible participants were informed of the purpose and procedures of the study, and consent was obtained before questionnaire administration. For participants unable to complete the questionnaire independently, investigators assisted by reading the questions aloud and recording answers. Questionnaires were checked on-site for completeness and logical consistency. For omitted responses or non-standard entries, promptly notify research subjects for correction. Exclude invalid questionnaires with incomplete responses, identical answers, or discernible response patterns. Two researchers independently entered the data, and inconsistencies were resolved by cross-checking with the original forms.

### 2.5 Measurement tools

#### 2.5.1 General information questionnaire

This form collected demographic and clinical data, including sex, age, education level, occupation, monthly income, marital status, place of residence, comorbidities, symptom presence, and sputum test results.

#### 2.5.2 Tuberculosis knowledge questionnaire

This 11-item questionnaire was developed based on national TB prevention guidelines. It covers core knowledge (e.g., disease nature, transmission routes, early signs, prevention, and policy awareness) and specific knowledge (e.g., treatment duration, sputum disposal, treatment precautions). Each correct answer scored 1 point; incorrect answers scored 0. A score ≥7 was classified as “adequate knowledge.” The scale was reviewed by TB experts and demonstrated acceptable internal consistency (Cronbach's α = 0.780) (31).

#### 2.5.3 Self-health management ability evaluation scale for chronic disease patients

Developed by Wang (32), this scale includes 49 items across five domains: cognitive ability, psychological adjustment, behavioral lifestyle, social environment, and treatment adherence. Each item is rated on a 5-point Likert scale (1 = never, 5 = always), with higher scores indicating better self-management. The Cronbach's α in this study was 0.919.

#### 2.5.4 Perceived social support scale (PSSS)

The 12-item PSSS, developed by Zimet et al. (33) and revised for Chinese populations by Jiang (34), evaluates support from family, friends, and others. Items are scored on a 7-point Likert scale, with total scores ranging from 12 to 84. Higher scores indicate greater perceived support. The scale had good internal reliability (Cronbach's α = 0.880).

#### 2.5.5 Hospital anxiety and depression scale (HADS)

The 14-item HADS, developed by Zigmond and Snaith (35), assesses anxiety and depression (seve items each). Each item is scored 0–3, with subscale scores categorized as normal (0–7), mild (8–10), moderate (11–14), and severe (15–21). This study used only the total score of the depression subscale to represent patients' depressive state. The HADS showed excellent internal consistency in this study (Cronbach's α = 0.919).

### 2.6 Statistical analysis

All statistical analyses were performed using IBM SPSS Statistics version 27.0 (IBM Corp., Armonk, NY, USA). A two-tailed *P* value < 0.05 was considered statistically significant. Descriptive statistics were used to summarize participant characteristics and scores of main study variables. Categorical variables were reported as frequencies and percentages, while continuous variables were assessed for normality using the Shapiro–Wilk test. As the main variables were not normally distributed, continuous variables were presented as medians with interquartile ranges (IQR: P25, P75).

Spearman's rank correlation analysis was conducted to examine the bivariate associations between TB knowledge, perceived social support, depression, and self-management ability.

To explore the mediating and chain-mediating effects of perceived social support and depression on the relationship between TB knowledge and self-management, a serial multiple mediation model was tested using the PROCESS macro version 3.5 for SPSS, developed by Hayes. Model 6 was specified to estimate the total, direct, and indirect effects.

The following regression paths were sequentially tested to examine the hypothesized mediation model: (1) the effect of TB knowledge on perceived social support (Path a1); (2) the effect of perceived social support on depression (Path d21); (3) the effects of TB knowledge and depression on self-management (Paths a2 and b2); and (4) the total and direct effects of TB knowledge on self-management after adjusting for the mediators. Bootstrapping procedures with 5,000 resamples were applied to estimate bias-corrected 95% confidence intervals (CIs) for all indirect effects. An indirect effect was considered statistically significant if the 95% CI did not include zero.

Three mediation pathways were evaluated: (1) a single mediation path from TB knowledge through perceived social support to self-management; (2) a single mediation path from TB knowledge through depression to self-management; and (3) a chain mediation path from TB knowledge through perceived social support and subsequently through depression to self-management.

The proportion of the total effect explained by indirect paths was also calculated to evaluate the explanatory power of the mediation model.

All mediation pathways and coefficients were visualized using a conceptual diagram generated by PROCESS output.

## 3 Results

### 3.1 Participant characteristics

A total of 204 patients with primary pulmonary TB were included in the analysis. The majority were male (54.4%) and 39.7% were aged 60 years or older. Most participants had a low education level, with 44.1% completing only elementary school or below. Regarding occupation, 43.1% were engaged in agricultural work, and 35.3% reported a monthly per capita income of ≤ 1,000 RMB. More than half of the patients (56.4%) tested positive in sputum smear or culture. Detailed demographic and clinical characteristics are presented in Table 1.

**Table 1:** The baseline characteristics of 204 patients with tuberculosis.

**Variables**	***n***	**%**
**Gender**		
Male	111	54.4
Female	93	45.6
**Age (years)**		
18–44	64	31.4
45–59	59	28.9
≥60	81	39.7
**Educational level**		
Primary school or below	90	44.1
Junior middle school	46	22.5
Senior high school and technical secondary school	30	14.7
College and above	38	18.6
**Occupation**		
Retirement	23	11.3
Unemployed	30	14.7
Organizational/Enterprise Employee	18	8.8
Laboring	19	9.3
Farming	88	43.1
Self-employed	12	5.9
Other	14	6.9
**Monthly per capita household income (RMB)**		
< 1,000	72	35.3
1,000–3,000	69	33.8
3,001–5,000	41	20.1
≥5,001	22	10.8
**Marital status**		
Unmarried/divorced/widowed	42	20.6
Married	162	79.4
**Residency**		
Urban	97	47.5
Rural	107	52.5
**Presence of symptoms**		
No	35	17.2
Yes	169	82.8
**Other chronic disease(number)**		
0	140	68.6
≥1	64	31.4
**Sputum smear or culture**		
Negatives	89	43.6
Positive	115	56.4

### 3.2 Variables scores

The total self-management score for 204 tuberculosis patients was 162.00 (148.00, 176.75); the knowledge score was 7.00 (5.00, 8.00), with an awareness rate of 51.5% (105/204); the perceived social support score was 61.00 (52.00, 72.00), and the depression score was 8.00 (5.00, 10.00), as shown in Table 2.

**Table 2:** Variables scores among 204 patients with tuberculosis.

**Projects**	**Number of items**	**Theoretical score range**	**Actual score [*M*(*Q_1_*, *Q_3_*)]**	**Average score per item [*M*(*Q_1_*, *Q_3_*)]**
Self-management	49	49–245	162.00 (148.00, 176.75)	3.31 (3.02, 3.61)
Tuberculosis knowledge	11	0–11	7.00 (5.00, 8.00)	0.64 (0.45, 0.73)
Perceived social support	12	12–84	61.00 (52.00, 72.00)	5.08 (4.33, 6.00)
Depression	7	0–3	8.00 (5.00, 10.00)	1.14 (0.71, 1.43)

### 3.3 Correlations of key variables

Correlation analysis using Spearman's method revealed that TB knowledge was positively correlated with perceived social support (*r* = 0.152, *P* < 0.05) and self-management (*r* = 0.257, *P* < 0.01), and negatively correlated with depression (*r* = −0.221, *P* < 0.01). Additionally, perceived social support was negatively correlated with depression (*r* = −0.321, *P* < 0.01) and positively correlated with self-management (*r* = 0.400, *P* < 0.01). Depression was negatively associated with self-management (*r* = −0.313, *P* < 0.01). Full results are presented in Table 3.

**Table 3:** Scores and correlation analysis of each study variable (*n* = 204).

**Variable**	**1**	**2**	**3**	**4**
Tuberculosis knowledge	1	–	–	–
Perceived social support	0.152^*^	1	–	–
Depression	−0.221^**^	−0.321^**^	1	–
Self-management	0.257^**^	0.400^**^	−0.313^**^	1

1, Tuberculosis knowledge; 2, Perceived social support; 3, Depression; 4, Self-management.

^*^P < 0.05; ^**^P < 0.01.

### 3.4 Tuberculosis knowledge and perceived social support in regression analysis

TB knowledge significantly predicted increased perceived social support (β = 0.827, *P* = 0.006) and decreased depression (β = −0.250, *P* = 0.009). Perceived social support negatively predicted depression (β = −0.105, *P* < 0.001), and positively predicted self-management (β = 0.606, *P* < 0.001). Depression, in turn, had a significant negative effect on self-management (β = −1.232, *P* = 0.007; Table 4).

**Table 4:** Regression analysis among the study variables.

**Predictor variable**	**Outcome variable**	***R* ^2^**	***F***	**β**	***t***	***P***	***LLCI***	***ULCI***
Tuberculosis knowledge	Perceived social support	0.036	7.586	0.827	2.754	0.006	0.235	1.419
Tuberculosis knowledge	Depression	0.154	18.274	−0.250	−2.653	0.009	−0.436	−0.064
Perceived social support				−0.105	−4.828	< 0.001	−0.148	−0.062
Tuberculosis knowledge	Self-management	0.194	16.097	1.227	1.992	0.048	0.012	2.441
Perceived social support				0.606	4.120	< 0.001	0.316	0.896
Depression				−1.232	−2.723	0.007	−2.125	−0.340

R^2^, coefficient of determination; F, F-statistic; β, standardized coefficients; t, t-statistic; P, P-value; LLCI, lower limit of the 95% CI (Confidence Interval); ULCI, upper limit of the 95% CI.

### 3.5 Mediation and chain-mediation analysis

Using TB knowledge as the independent variable, self-management as the dependent variable, and perceived social support and depression as mediators, a mediation model was tested with PROCESS (Model 6). The total effect of TB knowledge on self-management was statistically significant (β = 2.143, *P* < 0.01). When mediators were included, the direct effect remained significant (β = 1.227, *P* = 0.048), accounting for 57.26% of the total effect.

**Figure 1:**
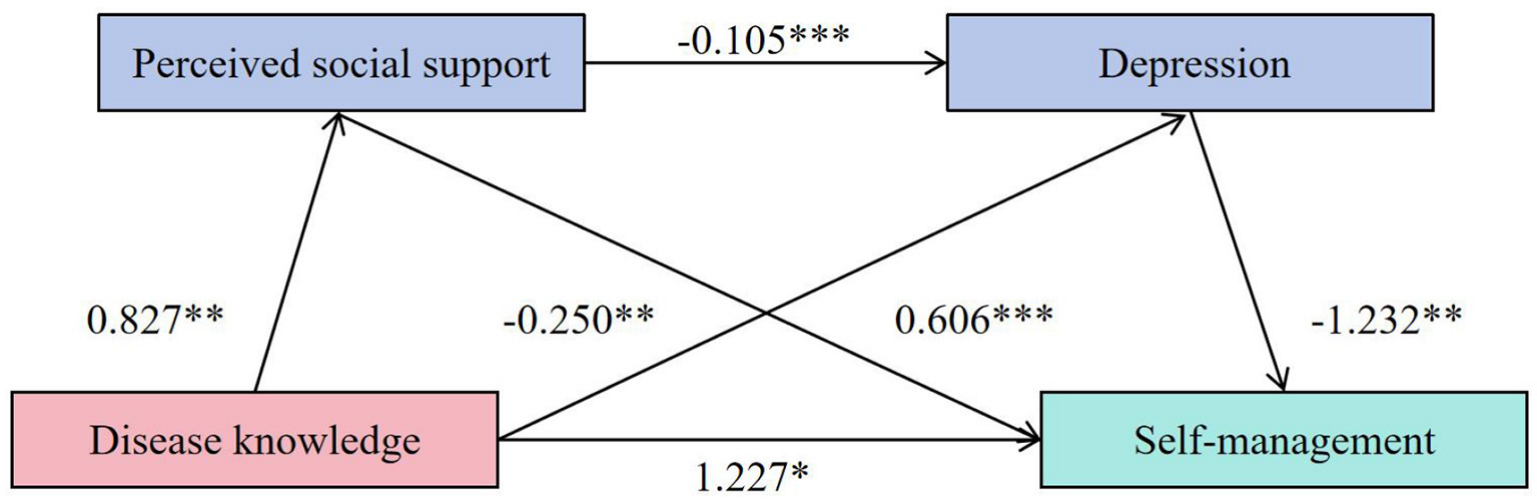
A chain mediation model to perceived social support, depression in the relationship between TB knowledge and self-management. *P < 0.05, **P < 0.01, ***P < 0.001.

Further analysis confirmed that all indirect effects were statistically significant. The total indirect effect was 0.916, accounting for 42.74% of the total effect. Specifically, the indirect effect via perceived social support alone was 0.501 (23.38%), via depression alone was 0.308 (14.37%), and via the chain path through both mediators was 0.107 (5.00%). None of the 95% bootstrap confidence intervals included zero, indicating the significance of all mediating paths (Table 5, Figure 1).

**Table 5:** Analysis of mediating effects.

**Item**	**Effect value**	**Boot SE**	**Bootstrap 95% CI**		**Percentage of relative effects (%)**
			***LLCI***	***ULCI***	
Total effect	2.143	0.641	0.878	3.407	
Direct effect	1.227	0.616	0.012	2.441	57.26
Total mediating effect	0.916	0.297	0.383	1.552	42.74
X → M_1_ → Y	0.501	0.232	0.118	1.031	23.38
X → M_2_ → Y	0.308	0.167	0.049	0.687	14.37
*X* → M_1_ → M_2_ → Y	0.107	0.058	0.016	0.241	5.00

X, tuberculosis knowledge; M_1_, perceived social support; M_2_, depression; Y, Self-management.

## 4 Discussion

This study aimed to examine the relationship between TB knowledge and self-management in patients with primary tuberculosis, focusing on the chain-mediating roles of perceived social support and depression. Grounded in the ABC-X family stress model, the research sought to elucidate the psychosocial mechanisms that influence patients' engagement in self-care behaviors. The findings confirmed that higher levels of TB knowledge were significantly associated with better self-management. More importantly, both perceived social support and depression acted as significant mediators—individually and sequentially—in this relationship. Specifically, TB knowledge positively influenced perceived social support, which subsequently reduced depressive symptoms, ultimately enhancing self-management behaviors. The chain-mediated effect accounted for 42.74% of the total influence of TB knowledge on self-management, indicating that the pathway from cognitive awareness to behavioral action is strongly modulated by psychosocial factors.

A notable contribution of this study lies in its integration of social and emotional mediators within a single explanatory model. Previous research has examined the impact of social support on tuberculosis outcomes separately (36), and Vestergaard et al. (24) demonstrated that depression among rheumatic disease patients is significantly associated with low self-management capacity. However, the relationship between depression and self-management in tuberculosis patients has not been previously reported, few studies have explored their combined effects and interactions. By applying the ABC-X model, this study offers a multidimensional understanding of the self-management process, highlighting how external resources and internal emotional states jointly promote health behaviors among TB patients. Unlike prior studies that assessed these variables separately (24, 36), we provide empirical evidence of their sequential interactions. This approach yields a more comprehensive understanding of how TB patients navigate treatment challenges, underscoring the importance of designing interventions that simultaneously address informational, emotional, and interpersonal needs.

The positive relationship between disease knowledge and self-management in TB patients has been widely acknowledged. Prior studies have consistently shown that improved TB knowledge enhances patients' awareness of symptoms, treatment regimens, and preventive strategies, thereby promoting adherence and encouraging health-promoting behaviors (4, 11, 17). This may be because individuals with higher levels of knowledge possess a more accurate and objective understanding of the symptoms, risks, and prevention methods associated with tuberculosis when confronting its threat. Their heightened awareness of disease-related risks prompts them to take timely measures to mitigate further physical and psychological harm, resulting in superior self-management capabilities. Previous studies have also indicated that patients' lack of proper understanding regarding tuberculosis control programs, the disease itself, and its treatment often leads to non-adherence to therapy (11). Hospitalization provides an optimal opportunity for patient education. This suggests that clinical healthcare providers should enhance tuberculosis knowledge education for patients, elevate their level of understanding, thereby improving self-management capabilities and promoting successful completion of the full course of treatment.

Perceived social support as an intermediary factor between disease knowledge and self-management indicates that patients with higher disease knowledge levels demonstrate better perceived social support and stronger self-management capabilities. As a chronic respiratory infectious disease severely threatening human health, tuberculosis patients often experience profound stigmatization, which reduces their willingness to cooperate with social support systems (37). Patients with higher knowledge levels can appropriately balance infection risks and resource utilization, enabling them to effectively leverage social support resources. Previous studies also indicate that low cultural literacy is a risk factor for perceived social support (38). Resource conservation theory posits that knowledge, as a unique energy source, helps individuals access support from conditional resources such as friendships, marriage, and power. Perceived social support is independently associated with better self-management. Perceived social support has been independently associated with better self-management. Patients with stronger support networks tend to report higher levels of treatment adherence, emotional stability, and health behavior maintenance (17, 18). The assistance provided by family and society—encompassing information, economic support, spirituality, life care, and treatment—motivates patients and enhances their ability to adopt beneficial self-management behaviors while avoiding maladaptive behaviors that could impede recovery. In designing interventions to improve TB patients' self-management abilities, incorporating social support from family and friends into assessment and education systems, alongside strengthening professional support from the medical team, can collectively enhance patients' perceived social support from multiple dimensions.

Likewise, depression is a prevalent psychological burden in TB patients, often leading to diminished self-care, poor adherence, and unfavorable clinical outcomes (20–23). Individuals with higher psychological wellbeing demonstrate better self-management of their illnesses (25). Li et al. (27) found in their study of hip replacement patients that individuals with higher levels of disease knowledge exhibited lower levels of depression. Further research is needed to explore the pathways through which self-management influences this population. Perceived social support and depression exert a chain-like mediating effect between disease knowledge and self-management. The lower the level of perceived social support among tuberculosis patients, the more severe their depression—a finding consistent with the research by Yohannes et al. (39). According to psychological stress and coping theory, individuals typically adopt one of two coping strategies when faced with disease-related stress: emotional-focused coping or problem-focused coping. When individuals lack sufficient personal resources, they are more likely to adopt emotional-focused coping, which often results in heightened anxiety and depressive emotions (40, 41). Social support can effectively compensate for the lack of these personal resources, helping patients to adopt problem-focused coping strategies, while also providing emotional value that alleviates the emotional toll of stress. Consequently, perceived social support has been identified as a protective factor against depression in TB patients, playing a critical role in overcoming treatment barriers and promoting treatment adherence (42, 43). In conclusion, higher levels of perceived social support and lower levels of depression buffer disease knowledge deficits and enhance self-management, suggesting a practical pathway for guiding comprehensive clinical interventions to improve self-management in TB patients.

The findings of this study can be further explained through the lens of the ABC-X family stress model. Within this framework, TB diagnosis acts as a stressor (A) that may overwhelm a patient's emotional and behavioral coping capacities. Knowledge serves as a key personal resource (B) that reduces uncertainty, enhances perceived control, and enables more informed responses to illness. Patients with adequate TB knowledge are more likely to recognize the importance of adherence, engage in preventative behaviors, and seek timely support.

Perceived social support also functions as a vital external resource (B). It not only provides instrumental assistance but also fosters emotional reassurance and reduces feelings of isolation. Greater social support enhances a patient's confidence in managing the disease and facilitates the mobilization of coping strategies. When patients feel supported, they are more likely to adhere to treatment, maintain follow-up visits, and avoid maladaptive behaviors. Depression is a cognitive-emotional response (C). When an individual possesses higher personal resources (B) and external resources (B), their emotional response (C) to stressors (A) is milder. Individuals with better mental health are more likely to adopt proactive health management behaviors (self-management-X) (44, 45). The Chinese Tuberculosis Control Guidelines (2021 Edition) state that clinical staff should regularly monitor patients' mental status, apply psychological knowledge and methods to provide emotional support, and bolster their confidence in recovery and daily life.

To date, no self-management intervention programs for tuberculosis patients based on the ABC-X family stress model have been identified. Based on the findings of this study, future interventions should integrate enhancing patients' disease knowledge and perceived social support, reducing negative emotions, and improving self-management capabilities into a comprehensive intervention. Compared to single measures, this integrated approach yields more significant, effective, and lasting improvements in self-management outcomes.

## 5 Limitations

This study has several limitations. First, its cross-sectional design prevents the establishment of causal relationships between TB knowledge, perceived social support, depression, and self-management. Longitudinal studies are needed to verify the directionality and temporal stability of the observed associations. Second, all data were collected using self-reported questionnaires, which may be subject to recall bias and social desirability bias, potentially leading to overestimation of knowledge or underreporting of psychological distress. Future studies should consider incorporating objective measures such as electronic adherence monitoring or clinician-assessed mental health tools. Third, the study was conducted in a single TB-designated tertiary hospital in Ningxia, which may limit the generalizability of the findings to other regions or healthcare settings. Multi-center studies involving more diverse populations are warranted. Fourth, the study focused only on TB knowledge, social support, and depression, while other relevant psychosocial variables—such as self-efficacy, perceived stigma, and health system accessibility—were not assessed. Including these in future models may provide a more comprehensive understanding of self-management determinants. Lastly, previous studies have examined the impact of demographic factors such as age, gender, educational attainment, occupation, income, marital status, number of dwellings, and place of residence on self-management among tuberculosis patients. This study did not control for the influence of these potential variables, which may introduce bias in the results. Future research should fully account for these factors.

## 6 Conclusion

This study demonstrated that TB knowledge significantly influences self-management, both directly and indirectly, through the sequential mediating roles of perceived social support and depression. These findings underscore the importance of integrating educational, social, and psychological interventions into TB care. For patients, improving disease knowledge enhances confidence and self-efficacy; for clinicians, assessing and supporting emotional wellbeing is essential for adherence; for health systems, developing multi-dimensional, person-centered TB management strategies may improve treatment outcomes and reduce public health burdens. The application of the ABC-X model offers a robust framework for understanding and optimizing TB self-management from a holistic perspective.

## Data Availability

The datasets presented in this article are not readily available because, due to the nature of this research, participants of this study did not agree for their data to be shared publicly, so supporting data are not available. Requests to access the datasets should be directed to HW: 1478597646@qq.com.
